# The preventive effect of *Brassica napus* L. oil on pathophysiological changes of respiratory system in experimental asthmatic rat

**Published:** 2013

**Authors:** Mehdi Kabiri rad, Ali Neamati, Mohammad Hossein Boskabady, Naser Mahdavi-Shahri, Maryam Mahmoudabady

**Affiliations:** 1 *Department of Biology, Faculty of Science, Mashhad Branch, Islamic Azad University, Mashhad, I. R. Iran*; 2 *Applied Physiology Research Centre and Department of Physiology, School of Medicine, Mashhad University of Medical Sciences, Mashhad, I. R. Iran *

**Keywords:** Airway Remodeling, Asthma, * Brassica napus*, Eozinophil, Inflammation, Rat, Sensitization

## Abstract

**Objective**: Asthma is an airway complex disease defined by reversible airway narrowing and obstruction, chronic airway inflammation, airway hyperresponsiveness, and tissue remodeling. The purpose of this study was to determine the effect of *Brassica napus* L. (*B. napus*) on airway pathologic changes in a rat model of asthma.

**Materials and Methods**: Twenty-four rats were divided into 4 groups: control, asthmatic, asthmatic treated with 0.5 mg/kg *B. napus* oil, and asthmatic treated with 0.75 mg/kg *B. napus* oil. To induce the experimental asthma, rats in groups 2, 3, and 4 received an i.p. injection of ovalbumin and aerosolized ovalbumin. Simultaneously, rats in groups 3 and 4 received *B. napus* oil daily by gavage. After 31 days, in all groups, thoracotomy was done and lung tissue samples were taken. For pathological evaluation, microscopic slides were prepared. The eosinophil numbers in the submucosal layer and thicknesses of smooth muscle layer of bronchioles were detected.

**Results: **Eosinophil numbers in the submucosal layer, as well as smooth muscle layer thicknesses were significantly lower in the rat group treated with 0.75 mg/kg *B. napus* oil as compared with asthmatic group (p<0.01, p<0.05).

**Conclusion:** *B. napus* could be useful as adjuvant therapy in rat model of asthma. This effect was probably related to its antioxidants components that reduce the levels of inflammatory mediators such as leukotrienes, IL-4, IL-5, and IL-13.

## Introduction

Asthma is an airway complex disease with reversible obstruction, airway hyperresponsiveness, inflammation, and remodeling of respiratory airways. The model includes structural changes of bronchial tree that can affect all layers. These changes include: epithelial metaplasia, hyperplasia of goblet cells and increase of mucus secretion, subepithelial fibrosis, increased smooth muscle mass due to hyperplasia and hypertrophy of cells, and increase in the number and diameter of the vessels. During the process of inflammation, migration of inflammatory cells, particularly eosinophils and mast cells to the submucosal and epithelium increase the number of these cells in bronchoalveolar lavage fluid (Martin and Tamaoka, 2006 ▶; Scheerens et al., 2002 ▶; Szelenyi, 2000 ▶). 

Among the inflammatory cells implicated in asthma, it is shown that activation of T helper 2 (Th2) causes airway inflammation and airway hyperresponsiveness. Activation of Th2 cells produces several cytokines such as interleukin (IL)-4, IL-13, IL-5, and IL-10. IL-4 and IL-13 elicit IgE production, IL-5 promotes eosinophil production in the bone marrow and IL-10 promotes B-cell differentiation into plasma cells (Cohn et al., 2004 ▶; Randolph et al., 1999 ▶).

Previous studies reported increased production of oxygen radicals and decreased antioxidant reserves such as glutathione, glutathione peroxidase, superoxide dismutase, vitamin C, and vitamin E in plasma and lavage fluid of asthmatic subjects. With increasing oxygen radicals, peroxidation of proteins, lipids, and nucleic acids a severe increase in responsiveness, secretion, and permeability of blood vessels occurs. In addition, free radicals activate inflammatory factors which result in exacerbation of allergic reactions (Murdoch and Lloyd, 2010 ▶; Charavaryamath, 2005 ▶). 

In this study the effects of *B. napus* L. (referred to as ‘canola’), on pathological changes of respiratory airways in a model of experimental asthma in rats were investigated. *B. napus* contains important antioxidants and omega-3 fatty acids, tocopherols (vitamin E), especially alpha- and gamma-tocopherol, polyphenols, and carotenoids such as carotene and xanthophylls. It is supposed to reduce inflammation and inflammatory mediators so that asthma symptoms alleviate (Elhiti et al., 2012 ▶; Fritsche et al., 2012 ▶; Horie et al., 2010 ▶; Xue et al., 2009 ▶).

## Materials and Methods

Twenty-four male and female Wistar rats of 3 month old, weighing approximately 200–250 g were used in the present study. Animals were housed in PVC cages under 12:12 light/dark cycle at 20±2 °C and were fed ad libitum with standard rat chow and tap water.

**Animal sensitization and animal groups**

Animals were sensitized to OA according to the method described previously (Schuster et al., 2000). Briefly, rats were sensitized to 1 mg OA (Sigma Chemical Ltd, UK) and 200 mg Al(OH)_3_ dissolved in 1 ml saline i.p. One week later they were given 1 mg OA and 200 mg Al (OH)_3_ dissolved in 1 ml saline i.p. as a booster dose. From day 14, sensitized animals were exposed to an aerosol of 4% OA for 18±1 days, 5 min daily. The aerosol was administered in a closed cylinder, measured 19 cm diameter and 16 cm high and 3 liter volume. Control animals were treated similarly but saline was used instead of OA solution. The study was approved by the ethical committee of the Mashhad Branch, Islamic Azad University, Mashhad, Iran.

 The animals were divided into 4 groups (n=6 for each group) as follow:

1- ''Control'' group.

2- ''Asthma'' group; an animal model of asthma.

3- Group ''Asthma+T 0.5'' which was treated with 0.5 mg/kg *B. napus* oil for 31 days after sensitization.

4- Group ''Asthma+T 0.75'' which was treated with 0.75 mg/kg *B. napus* oil for 31 days after sensitization.

The net oil of *B. napus* was provided from Ladan Company, Iran.

**Tissue preparation and histopathological analysis**

At the end of the protocol, the animals were killed by i.p. administration of ketamine/xylazine. Lungs were fixed in formalin and embedded in paraffin. Tissue sections (5 µm) were stained with hematoxylin-eosin and examined under light microscopy to evaluate the eosinophil numbers in the submucosal layer and thicknesses of smooth muscle layer of bronchioles. 

The thicknesses of smooth muscle layer were measured in 8 points of 2-3 bronchiole ducts and the mean was reported. The number of eosinophils in submucosal layer of each bronchiole duct was counted and to determine it in 100 µm of submucosal layer the following formula was used:

Bronchiole duct circumference = π × bronchiole duct diameter Number of eosinophils in 100 µm of submucosal layer = number of eosinophils in submucosal layer × 100 / bronchiole duct circumference in submucosal layer.

**Statistical analysis**

All data were expressed as mean±SEM. Comparisons were performed using one-way ANOVA with SPSS software. *P*-values less than 0.05 were considered to be statistically signiﬁcant.

## Results

**Figure 1:**
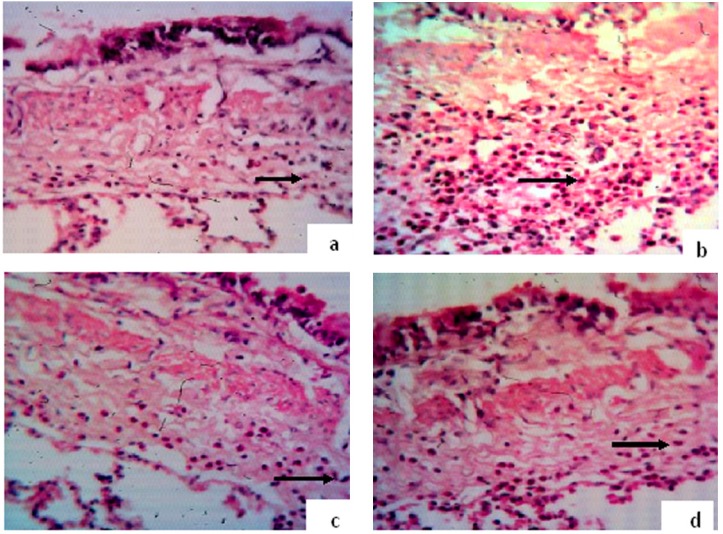
Hematoxylin-eosin staining (magnification ×400). Eosiniphil cells (black arrows) in 100 µm of submucosal layer of bronchioles in (a) Control group (b) Asthmatic group (c) ''Asthma+T 0.5'' (d) ''Asthma+T 0.75''.

**Figure 2:**
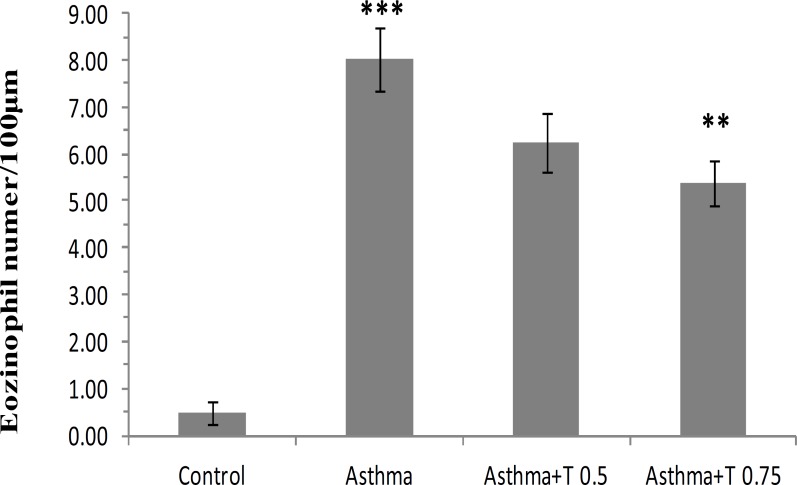
Number of eosinophils in 100 µm of submucosal layer of bronchioles in different groups of study. Data are expressed as mean±SEM,. (n = 6). **p<0.01, versus Asthma group. ***p< 0.001, versus Control group.

The number of eosinophils in submucosal layer of bronchioles in asthmatic group of rats was significantly increased compared with control group. This parameter in the groups treated with *B. napus* oil was decreased compared with asthmatic group (Figure 1). The reduction in ''Asthma+T 0.75'' group was significant (p<0.01, Figure 2).

**Figure 3:**
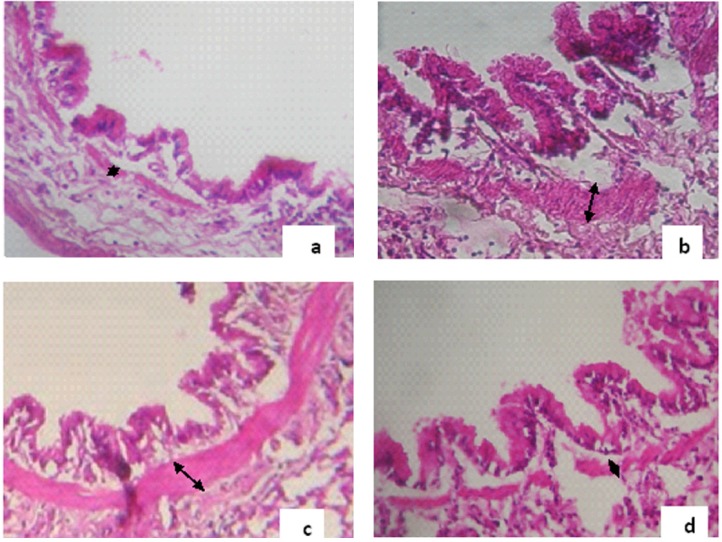
Hematoxylin-eosin staining (magnification ×400). Smooth muscle layer thickness (black arrows) of bronchioles in (a) Control group (b) Asthmatic group (c) ''Asthma+T 0.5'' (d) ''Asthma+T 0.75''.

**Figure 4:**
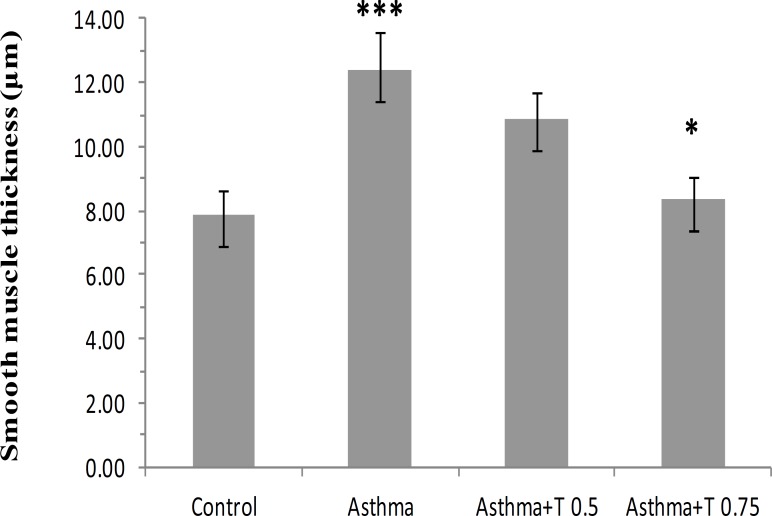
Thickness of smooth muscle layer of bronchioles in different groups of study. Data are expressed as mean±SEM. (n = 6). *p<0.05 versus Asthma group, ***p<0.001 versus Control group

The thickness of smooth muscle layer of bronchioles in asthmatic group of rats was significantly increased compared with control group. This index in the groups treated with *B. napus* oil was decreased compared with asthmatic group (Figure 3). The reduction in ''Asthma+T 0.75'' group was significant (p<0.05, Figure 4).

## Discussion

The results of this study showed the number of eosinophils in 100 µm of submucosal layer as well as the thickness of smooth muscle layer of bronchioles in groups treated with *B. napus* oil was less than asthmatic group. 

This protective effect of *B. napus* oil was more visible in higher dose. Since asthma is a common inflammatory disease of respiratory system (Zhang et al, 2011; Cohn et al., 2004 ▶; Randolph et al., 1999 ▶), anti-inflammatory drugs are the mainstay therapy in this condition (Hakim et al, 2012 ▶; Higashi et al, 2012 ▶).

There are different documents which show anti-oxidant and anti-inflammatory properties of *B. napus* (Elhiti et al., 2012 ▶; Fritsche et al., 2012 ▶; Horie et al., 2010 ▶; Xue et al., 2009 ▶). As the present study showed, *B. napus* reduced the eosinophil numbers in the submucosal layer, as well as smooth muscle layer thicknesses of bronchioles in sensitized rats so it can confirm the anti-inflammatory effect of this plant. According to investigations, the most important inflammatory mediators implicated in the pathogenesis of asthma include:

1. IL-3: involves in the production of IgE and initiating of inflammation, increases the thickness of smooth muscle layer by smooth muscle cell hyperplasia (Ma et al., 2007 ▶).
2. IL-4: involves in the production of IgE and initiation of inflammation (Murdoch and Lloyd, 2010 ▶).
3. IL-5: increases both production of eosinophils in the bone marrow and activation of them. Activated eosinophils release lipid mediators including leukotrienes, prostaglandins, and platelet-activating factors similar to mast cells and basophils (Halwani et al., 2010 ▶).
4. Leukotrienes: cause bronchospasm, airway wall edema, excess mucus secretion, increase vascular permeability, and migration of inflammatory cells (Simopoulos, 2009 ▶).

Moreover, it was shown that antioxidants such as omega-3, vitamin E, α-lipoic acid, and mepacrine reduce leukotrienes, IL-4, and IL-13. As Broughton showed in 1997 with a population of asthmatics, omega-3 fatty acids decreased leukotrienes production (Broughton et al., 1997 ▶). This reduction was mediated by blocking of arachidonic acid metabolism (Simopoulos, 2009 ▶). In 1988, Hashimoto and colleagues demonstrated increased linolenate (omega-3) to linoleate (omega-6) ratio in diet decreased leukotrienes (Hashimoto et al., 1988). In 2002, Li-weber and colleagues showed that vitamin E inhibited IL-4 gene expression in peripheral T cells (Li-Weber et al., 2002 ▶).

 One study reported that γ-tocopherol reduced inflammation by inhibiting inflammatory mediators such as prostaglandin and leukotrienes and reducing tumor necrosis factor (TNF) (Jiang and Amesb, 2003 ▶). Ram and colleagues in 2008 showed in a mouse-model of asthma that mepacrine (as antioxidant) decreased inflammation and airway hyperresponsiveness and reduced significantly the amount of IL-4, IL-5, IL-13, IgE, leakage of inflammatory cells, and the number of eosinophils in bronchoalveolar lavage fluid (Ram et al., 2008 ▶). In 2011, Boskabady and colleagues demonstrated that in sulfur mustard exposed guinea pigs, vitamin E significantly reduced response reaction, the amount of white blood cells (WBC) particularly eosinophils, and IL-4 (Boskabady et al., 2011 ▶). 

Above mentioned studies show the effects of antioxidants on inflammatory mediators and confirm our results about eosinophils. Moreover, omega-3 and vitamin E prevent oxidation of fatty acids in membrane lipids thus have anti-inflammatory effects by stabilizing cell membranes (Sagols and Priymenko, 2011 ▶). In the other hand, vitamin E regulates T lymphocyte activity and inhibits the production of IgE (Park et al., 2010 ▶).

As *B. napus* oil contains high levels of antioxidants such as omega-3 and vitamin E, it can reduce necrosis and migration of eosinophils as well as inhibits smooth muscle cell hyperplasia in the walls of respiratory tract by decreasing the amount of leukotrienes, IL-13, and IL-4.* B. napus* oil improves some symptoms of experimental asthma in rats. These effects are likely due to its antioxidants including omega-3, vitamin E (tocopherol), polyphenols, and carotenoids which reduce the mediators of inflammation and inflammatory cytokines such as IL-4, IL-13, and leukotrienes. 

In conclusion the results of the present study suggest *B. napus* could be useful as adjuvant therapy in rat model of asthma. This effect was probably related to its anti-inflammatory and antioxidants components.
